# Metabolic hormones are integral regulators of female reproductive health and function

**DOI:** 10.1042/BSR20231916

**Published:** 2024-01-31

**Authors:** Faria Athar, Muskan Karmani, Nicole M. Templeman

**Affiliations:** Department of Biology, University of Victoria, Victoria, British Columbia V8P 5C2, Canada

**Keywords:** hypothalamic-pituitary-ovarian axis, insulin, metabolic disorders, nutrient-sensing, obesity, polycystic ovary syndrome (PCOS)

## Abstract

The female reproductive system is strongly influenced by nutrition and energy balance. It is well known that food restriction or energy depletion can induce suppression of reproductive processes, while overnutrition is associated with reproductive dysfunction. However, the intricate mechanisms through which nutritional inputs and metabolic health are integrated into the coordination of reproduction are still being defined. In this review, we describe evidence for essential contributions by hormones that are responsive to food intake or fuel stores. Key metabolic hormones—including insulin, the incretins (glucose-dependent insulinotropic polypeptide and glucagon-like peptide-1), growth hormone, ghrelin, leptin, and adiponectin—signal throughout the hypothalamic–pituitary–gonadal axis to support or suppress reproduction. We synthesize current knowledge on how these multifaceted hormones interact with the brain, pituitary, and ovaries to regulate functioning of the female reproductive system, incorporating *in vitro* and *in vivo* data from animal models and humans. Metabolic hormones are involved in orchestrating reproductive processes in healthy states, but some also play a significant role in the pathophysiology or treatment strategies of female reproductive disorders. Further understanding of the complex interrelationships between metabolic health and female reproductive function has important implications for improving women’s health overall.

## Close ties between nutritional status and female reproductive function

Reproduction is an energetically expensive process, and the energetic costs are largely borne by the females of many species. The mammalian female reproductive system is responsible for producing female gametes, facilitating their fertilization with sperm, supporting embryonic-fetal growth and development, and enabling the birth and nourishment of the offspring. These complex processes require a high level of communication between various organ systems, and so reproduction is under tight control of centrally produced hormones released by the hypothalamus and pituitary, as well as signaling factors produced by the placenta, developing embryo, and tissues of the reproductive system. Considering the high energetic requirements of gamete production, gestation, and lactation, it is clear that levels of food and energy stores are additional pieces of information that must be incorporated into the control of reproductive processes. Therefore, hormones that are classically defined by their metabolic roles are also critically important for regulating reproduction, including those which relay acute changes in ingestion and nutrient levels (e.g*.*, insulin, the incretins, growth hormone, and ghrelin), as well as hormones that communicate stored metabolic fuel levels (e.g*.*, leptin and adiponectin).

Strong links between nutritional status and female reproductive function are evident throughout the animal kingdom. Vertebrate orexigenic and anorectic neuropeptides that are classified based on their effects on appetite also modify levels of gonadotropin hormones in the reproductive axis [1]. Many bird species breed seasonally, which restricts reproductive activity to periods when local food supply is optimal for supporting increased metabolic demands [2,3]. Food availability affects fecundity and the timing of sexual maturity in fishes, and female iteroparous fishes may skip a spawning season if nutrient levels are insufficient [4,5]. There are also insect and nematode species that can enter diapause states to reversibly suspend development and reproduction under unfavourable conditions. Entry into or recovery from diapause in these invertebrate organisms is orchestrated in part by evolutionarily conserved signaling systems that communicate nutritional status [6,7]. Mammals exhibit patterns of sexual dimorphism that are consistent with the concept that females are better suited for withstanding periods of food scarcity, which would increase chances of reproductive success in nutritionally-fluctuating environments [1]. For instance, in contrast with males, female mammals tend to favor energy storage over the capacity for rapid fuel mobilization, and are more prone to accumulate adipose mass in subcutaneous depots [8–10].

In humans, the relationship between nutrient levels and female reproductive health is most obvious when an imbalance in nutrient and energy levels pushes functioning of the reproductive system off-kilter. This is exemplified by physiological responses to food restriction and excessive energy expenditure, or conversely by the reproductive system disorders that are linked to overnutrition.

### Inadequate energy and reproductive dysfunction

Energy deficiency caused by stress, low food intake, or strenuous exercise can result in a suppression of neuroendocrine signals that allow normal menstruation and ovulation. This deregulation is signified by a loss or alteration of gonadotropin hormone pulsatility, and leads to ovarian responses such as decreased estradiol production [11–13]. Low energy availability can thereby cause primary amenorrhea, a delay in menarche, or secondary amenorrhea, a temporary halt in natural menstrual cycling [14]. Studies in human populations exemplary of a negative energy balance have shed light on aberrant menstrual patterns. Ballet dancers [15,16] and athletes [17,18] engaged in high physical activity may have a delayed pubertal onset. Additionally, disordered eating, excessive exercise, or lifestyle stressors can cause menses loss in erstwhile normal-ovulatory women [19–22]. Temporary food deprivation or fasting can also induce a drop in gonadotropin levels and rise in cortisol [23–27]. Furthermore, women in poverty-stricken and/or strenuous labor-demanding societies experience increased risks of adult amenorrhea and lower birth rates [28–32].

Importantly, the ready availability of energy or metabolic fuels is more critical to reproductive fitness than adiposity *per se*. Food-deprived or over-exercised females can adjust food intake or activity to restore normal ovarian cyclicity and gonadotropin pulsatility before changes in adiposity or weight are evident [33–36]. Similarly, short fasting intervals halt ovarian cycles in Syrian hamsters without affecting adiposity, by reducing free fatty acid oxidation [37,38]. Maintaining glucose availability also preserves reproductive function in rodents and primates [39–43].

Amenorrhea and subfertility are not merely disorders of the reproductive system, but instead have broad physiological impacts. Just as a reduction in estrogen levels in postmenopausal women increases risks of cardiovascular disease [44], bone frailty [45], and neuropsychiatric disorders [46], a premenopausal estrogen deficiency caused by amenorrhea leads to compromised cardiovascular, skeletal, and mental health [47–51]. Thus, reproductive system responses to nutritional cues have far-reaching effects.

### Excess energy and reproductive dysfunction

Undernutrition can suppress signals that allow reproduction, but chronic overnutrition is also associated with reproductive dysfunction. The recent prevalence of ultra-processed and low-satiating foods, combined with a more sedentary lifestyle, has led to a surplus of calories in everyday life [52–56]. Animal studies have shown that high-energy, high-fat diets interfere with reproductive function independent of obesity [57–60]. Additionally, obesity and a high body mass index are themselves associated with precocious menarche [61–65], menstrual cycle irregularities [66–69], infertility [70–73], miscarriage [74–76], and fetal abnormalities [77–82]. Diets that are high in refined carbohydrate also predict an earlier age of menopause [83,84].

Energy overload and reproductive dysfunction are also closely associated in the context of polycystic ovary syndrome (PCOS), the most prevalent female reproductive disorder [85,86]. The diagnostic features of PCOS include hyperandrogenism, menstrual cycle irregularities, and an accumulation of fluid-filled cysts in the ovaries [87,88]. However, PCOS has heterogeneous symptoms that often include obesity, elevated insulin, and/or a diminished capacity for glucose disposal [89,90]. These metabolic characteristics exacerbate the reproductive features of PCOS through such means as heightening testosterone levels [91–95]. Hypercaloric, high-fat diets also potentiate the traits of PCOS [96–98]. Women with PCOS tend to have difficulty conceiving, as well as a greater risk of pregnancy complications such as gestational diabetes, preeclampsia, or miscarriage [99–109]. PCOS is also associated with increased incidence of Type 2 diabetes [110–112], hypertension [110,113–117], high cholesterol [118–120], stroke [121,122], and cancer [123–126].

Weight loss [127–129], exercise [129–134], and bariatric surgery to limit food intake [135–140] are useful clinical tools for treating some aspects of the reproductive dysfunction associated with energy surplus. Lifestyle approaches such as balanced diet selections favoring whole grains, vegetables, fish, and unsaturated fats rather than saturated or trans fats are associated with increased fertility, improvements to PCOS symptoms, and beneficial impacts on other aspects of gynecologic health [141–144]. However, there is a lack of widespread awareness that diet has important implications for reproductive health (beyond effects on weight loss or perinatal health), and this is compounded by barriers preventing equal access to healthy diet options [143].

**Figure 1:**
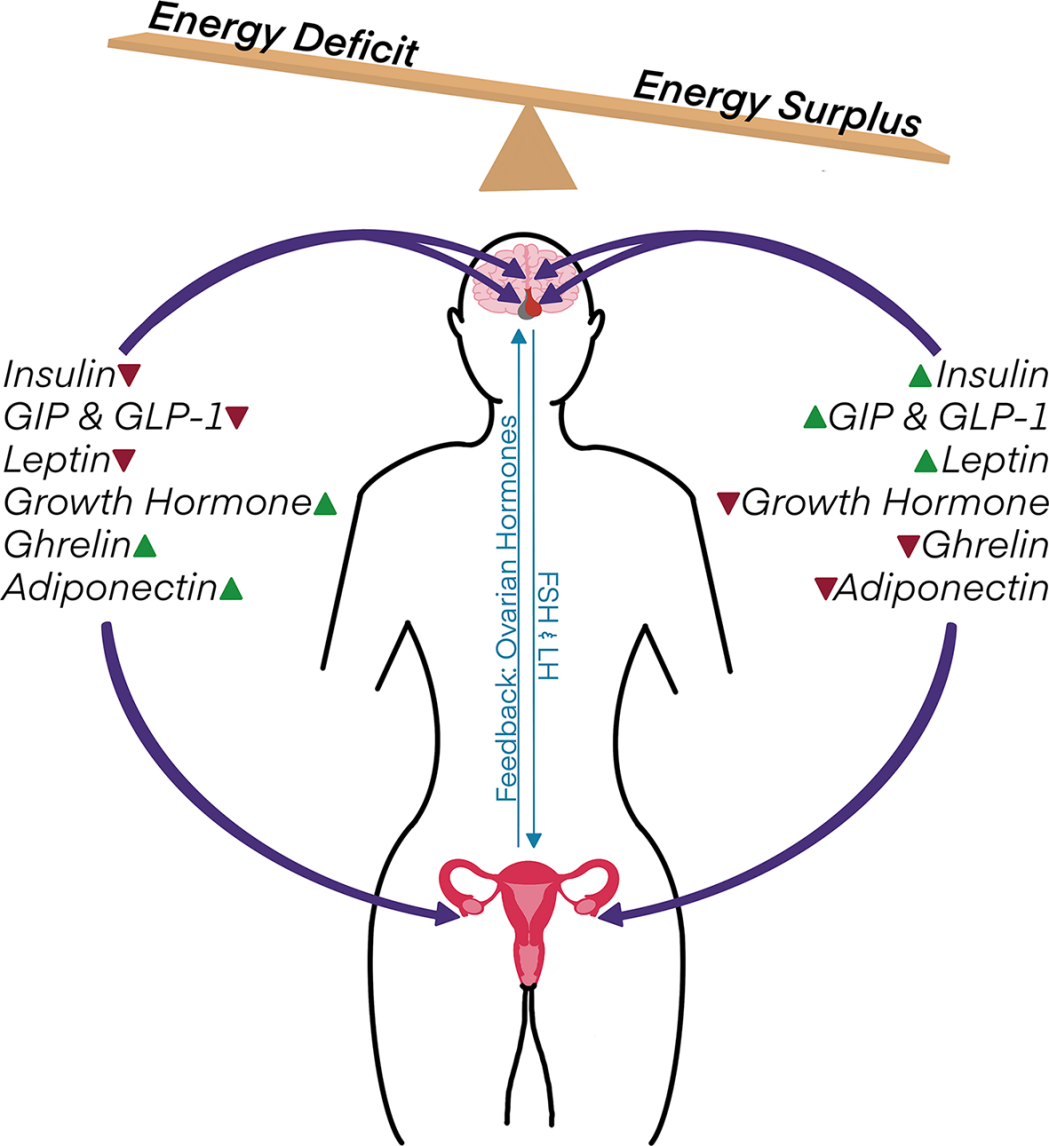
Metabolic hormones act as key intermediaries in linking nutrient and energy status to female reproductive function Energetic deficits generally decrease levels of insulin, the incretin hormones (GIP and GLP-1), and leptin while also raising growth hormone, ghrelin, and adiponectin. Conversely, food ingestion and/or a chronic energy surplus causes the opposite shift in circulating levels of these hormones. Metabolic hormones act directly within the hypothalamus, pituitary, and ovaries to modulate reproductive processes. Their effects are thereby integrated into the reproductive axis, in which the hypothalamus and anterior pituitary communicate with the female reproductive system through the gonadotropins follicle-stimulating hormone (FSH) and luteinizing hormone (LH), while the ovaries in turn provide feedback via steroid hormones and other signaling factors.

Effective management of nutritional and energy inputs is imperative for maintaining reproductive health. Nutrition and energy balance affect many aspects of reproductive health, including the menstrual cycle, fertility, pregnancy, fetal health, and age-related reproductive decline. This is due in part to the hormones that interpret food intake and fuel stores, which cooperate with cellular nutrient sensors to trigger the appropriate physiological responses for systemic energy homeostasis. These metabolic hormones engender changes across the reproductive axis, from the hypothalamus and pituitary to peripheral tissues of the female reproductive system (Figure 1).

## Basic regulation of female reproduction

### The hypothalamus and anterior pituitary

The hypothalamic–pituitary–gonadal (HPG) axis comprises a system wherein the hypothalamus and anterior pituitary cooperate to centrally control gonadal maturity and function. Gonadotropin-releasing hormone (GnRH) is a tropic hormone secreted by a small subset of hypothalamic neurons in response to a suite of peripheral signals and neuronal messengers, including inputs from kisspeptin (Kiss1) neurons, astrocytes, γ-aminobutyric acid (GABA) neurons and pro-opiomelanocortin (POMC) neurons [145,146]. Pulses of GnRH released into portal circulation range in frequency from pulsatile to surge mode, depending on sex, age, and menstrual cycle phase [147–150]. Although the HPG axis is first established *in utero*, it is largely silenced until the initiation of nocturnal GnRH pulses during the onset of puberty [151,152]. Kisspeptin signaling is a key player in reactivating the HPG axis and initiating the pulsatile hypothalamic GnRH secretion required for sexual maturity and reproductive function [153–157]. Thereafter, rhythmic changes in frequency and amplitude of GnRH pulses are integral for controlling the differential secretion pattern of the two gonadotropin hormones, together with regulatory input by other systemic and paracrine factors.

Gonadotroph cells of the anterior pituitary produce the gonadotropins, luteinizing hormone (LH) and follicle-stimulating hormone (FSH) [158,159]. GnRH signaling induces differential expression of genes encoding LHβ and FSHβ subunits, in addition to regulating LH and FSH exocytosis [160]. In turn, FSH and LH exert controls over ovarian function, including steroidogenesis, follicular development, and ovulation. Together, forward-acting and feed-back regulatory loops facilitate a dynamic, nuanced reproductive axis that can be adjusted at multiple levels in response to internal and external conditions [161]. For instance, crucial information related to nutritional status and energy balance is incorporated into the reproductive axis via metabolic hormones exerting effects on GnRH production and release, gonadotropin secretion, and ovarian functions.

### The ovaries

Oogenesis, folliculogenesis, and the production of steroid hormones and other signaling factors are tightly regulated ovarian processes that are responsive to inputs such as metabolic hormone signaling. Oogenesis begins early in embryonic development with primordial germ cells that divide by mitosis to form oogonia, which may continue to mitotically divide, undergo programmed cell death, or enter into meiosis as primary oocytes [162]. Primary oocytes do not complete meiosis, but are instead arrested and individually surrounded by a sheath of granulosa cells in structures called primordial follicles [163,164]. Female humans are born with approximately one million non-atretic primordial follicles, which constitute the initial ‘ovarian reserve’ [165].

After birth, primordial follicles are continuously recruited into a pool of growing follicles that routinely undergo atresia, or apoptosis-mediated degeneration [166–169]. Since primordial follicles lack an independent blood supply, the early stages of folliculogenesis are under limited endocrine regulation; the transition from primordial to primary follicle is largely controlled by intra-ovarian paracrine signaling [170]. Granulosa cells of a primary follicle begin to express FSH receptors, and FSH is involved in stimulating the progression into a secondary follicle [171–173]. Theca cells enveloping the follicle develop LH receptors late in the secondary follicle stage [174], and the thecal layer becomes increasingly vascularized as the antral follicle develops [175]. Preantral stages of folliculogenesis continuously generate a pool of developing follicles which almost all default to atresia [170,176]. However, with the stimulation by gonadotropins that occurs after puberty, a very small cohort of ∼10 antral follicles is recruited each month for further maturation [176].

In response to FSH and LH, granulosa cells and theca cells cooperatively participate in steroidogenesis to produce androgens, estrogens, and progesterone [177,178]. The dominant follicle heightens production of estradiol and other signaling factors that act locally as well as centrally suppressing FSH production; this ultimately pushes the non-dominant follicles to atresia, since they have fewer FSH receptors and are outcompeted for FSH, a survival factor that inhibits follicular atresia [179,180]. The oocyte resumes meiosis as the dominant antral follicle continues to mature. Eventually, sustained high estradiol levels triggers a surge in centrally produced LH and FSH, driving follicle rupture and ovulation of the oocyte surrounded by supporting granulosa cells called cumulus cells [181,182]. Meanwhile, the residual follicle somatic cells differentiate into a temporary endocrine gland called the corpus luteum [183,184]. Ovulation occurs monthly in healthy females until approximately 50–55 years of age, when the ovarian reserve is exhausted [185–188]. The resultant drop in levels of follicle-produced hormones and signaling factors ripples through the HPG feedback system, leading to fluctuations in GnRH pulses and high circulating LH and FSH before gonadotropin levels eventually decline after menopause [189,190].

### Cellular nutrient sensors

Cellular nutrient sensors are expressed in the hypothalamus, pituitary and ovaries, where they interpret local quantities of metabolites or energy-carrying molecules and interact with different cellular players to govern functioning of the female reproductive system. As comprehensively described in recent review articles [191–195], these nutrient sensors exert direct and indirect effects on female reproductive function. For instance, mechanistic target of rapamycin (mTOR), a protein kinase that promotes anabolic processes in response to increased amino acids and growth signals, is involved in regulating primordial follicle activation and granulosa cell proliferation, among other metabolically important functions. Similarly, AMP-activated protein kinase (AMPK), a nutrient sensor activated by a drop in cellular energy, plays a role in maintaining organismal energy homeostasis that extends to influencing pubertal timing and oocyte maturation.

Along with their own nutrient-sensing functions, cellular nutrient sensors are integral signaling intermediaries by which metabolic hormones probably direct some of their effects on reproductive physiology. For example, adiponectin can induce mTOR inhibition and AMPK activation [196], whereas the insulin signaling cascade is capable of promoting mTOR activity and inhibiting AMPK [197,198]. However, delineating these relationships in the context of reproductive functions is complicated by tissue-specific and context-dependent interactions. For instance, the adiposity signal leptin inhibits hypothalamic AMPK but activates skeletal muscle AMPK, whereas fasting-induced ghrelin activates AMPK in the hypothalamus while inhibiting AMPK in adipose tissue and liver [199]; it is unclear how these signaling hubs interact elsewhere, such as in ovarian cells. Intricate, bidirectional cross-talk between signaling pathways of cellular nutrient sensors and metabolic hormones further exacerbates the difficulties of defining their individual roles in regulating reproductive processes.

## Insulin

Insulin is best known for maintaining blood glucose levels, but it also regulates carbohydrate, lipid and protein metabolism, appetite, cell division, cell growth, and lifespan. A peptide hormone predominantly produced by β cells of the pancreatic Islets of Langerhans, insulin is secreted in response to the glucose, fatty acids, and amino acids that become elevated in circulation due to food intake. However, insulin levels are under multifactorial control, and autonomic nervous system innervation as well as other hormones (such as growth hormone and glucagon-like peptide 1) also affect insulin production and secretion; circulating levels are further controlled at the level of its clearance [200–203]. Insulin was discovered in 1921-22 with the extraction and purification of a pancreatic substance that could effectively lower blood glucose levels in patients with Type 1 diabetes [204,205]. It had a transformative impact on the treatment of diabetes, establishing its fundamental metabolic role.

Although insulin was discovered in mammals, it is now well known that insulin-like peptides and their highly conserved signaling cascades regulate metabolism, development, and aging across the animal kingdom. Ligand binding to insulin/insulin-like growth factor 1 (IGF-1) tyrosine kinase receptors leads to the activation of downstream signaling effectors, including phosphatidylinositol 3-kinase (PI3K, whose activities are counteracted by phospholipid phosphatases like PTEN) and the serine/threonine kinase AKT. The PI3K/AKT signaling pathway is associated with promoting glucose uptake and storage, suppressing hepatic glucose release, stimulating lipogenesis, and inhibiting mobilization of stored lipids [197,206]. Another major branch of insulin/IGF-1 signaling is transduced via the mitogen-activated protein kinase (MAPK)/ERK cascade, which is primarily involved in regulating growth and cell proliferation [197,206]. These signaling cascades also interact with other nutrient sensor mechanisms, through such means as activating mTOR and inactivating AMPK [197].

Lowering insulin below a critical threshold causes diabetes, but elevated insulin is also associated with detrimental changes. Insulin hypersecretion is a driving factor for insulin resistance, obesity, and other aspects of metabolic dysfunction [207]. Notably, in the ‘insulin resistant’ state that is defined by impaired insulin-induced glucose disposal, only a subset of insulin-regulated processes have diminished responses to insulin; tissues such as ovaries and the pituitary might remain mostly insulin-responsive [207–209]. The elevation in circulating insulin that often accompanies insulin resistance could thereby exacerbate insulin signaling responses in the female reproductive system.

### Insulin and the female reproductive system

Insulin and the insulin signaling pathway are important regulators of reproduction, and there can be detrimental outcomes of either insufficient or excess levels. Manipulations such as brain-wide deletion of insulin receptors (InsR) suppress GnRH release in mice, leading to impaired follicle maturation and reduced fertility [210]. On the other hand, high insulin levels induced by high-fat feeding are accompanied by fewer estrous cycles, fewer preantral and antral follicles, and smaller litters; exogenous insulin also causes a reduction in murine oocyte yield and quality [198,211,212]. In humans, an infusion of insulin and lipids acutely suppresses FSH and LH levels [213]. Elevated insulin at birth and during childhood is associated with earlier puberty [214], and insulin levels in reproductive-aged women are negatively correlated with levels of anti-müllerian hormone (AMH), an ovary-produced hormone that signifies ovarian reserve [215].

Insulin receptors are expressed widely in the brain [216,217], including in GnRH neurons, astrocytes, and Kiss1 neurons [218–221]. Astrocytes are implicated in the insulin-mediated regulation of GnRH release [222], and InsR ablation in astrocytes results in altered ovarian cycling, impaired oocyte maturation, hypogonadism, and subfertility [223]. Cultured GnRH cell-lines also contain InsRs [224], and insulin stimulates GnRH expression and promotes its effects on gonadotropin secretion *in vitro* [225–227]. GnRH-specific InsR knockout mice are protected from obesity-associated infertility, with GnRH pulses that are comparable to lean control mice [228]. InsR knockout in the pituitary [211] or ovarian theca cells [229] also protects female mice from high-fat diet-induced infertility, showing that elevated insulin acts across multiple systems to impair reproductive function under conditions of nutrient surplus.

Insulin has direct ovarian effects on metabolism, steroidogenesis, and folliculogenesis. The InsR is expressed in oocytes, granulosa cells, and theca cells of rodent, bovine, and human ovaries [230–234]. Insulin signaling is crucial for supporting glucose uptake and glycolysis in the ovary, to provide energy for folliculogenesis [235,236]. Insulin also plays an important role in steroidogenesis, by cooperating with LH to stimulate androgen production in theca cells [91,237,238]. It promotes the primordial to primary follicle transition in a rat ovarian organ culture system [239], and supra-physiological levels of insulin stimulate bovine oocyte cleavage, maturation and meiotic progression *in vitro* [240,241]. *In vivo*, oocyte-specific InsR deletion appears to have minimal impacts on fertility in mice [242]. Similarly, InsR ablation in murine granulosa or theca cells can lead to altered steroidogenesis and gene expression changes without overt effects on gross ovarian morphology or fertility under standard dietary conditions [229,243]. However, insulin and IGF-1 are closely related and can bind to each other's receptors or hybrid insulin-IGF-1 receptors with varying affinities [244]. Notably, double knockout of InsR and IGF-1 receptors in granulosa cells causes significant infertility, by impairing oocyte development and ovulation to a greater degree than knockout of either receptor alone [243]. IGF peptides, binding proteins, and receptors are expressed in human follicles [245], and IGF-1 is itself important for regulating follicular growth and survival as well as FSH-induced processes such as estradiol production, granulosa cell differentiation, and ovulation [246–252].

Downstream insulin/IGF-1 signaling components are established players in maintaining ovarian function and balance in folliculogenesis. For instance, overexpression of PI3K in the oocytes of neonatal mice increases follicular numbers, reduces apoptosis, and triggers an anovulatory state due to an excess of overgrown follicles [253]. Similarly, oocyte-specific removal of the counter-regulatory PTEN causes premature activation and exhaustion of the quiescent follicular pool [254]. Akt is widely expressed in ovarian stromal and germ cells in humans [255] and rodents [256], and Akt-deficient mice have delayed puberty onset, reduced fertility, altered steroid hormone levels, and a predisposition for PCOS-like phenotypes [257,258]. PI3K/AKT signaling maintains the primordial follicle pool in part by phosphorylating and inactivating the FOXO3 transcription factor, which otherwise suppresses primordial follicle activation when active [259]. Mice with constitutively activated oocyte FOXO3 maintain follicle numbers, gonadotropin levels, and youthful gene expression profiles with advancing age [260].

Abnormal insulin levels—relatively common with metabolic disorders—are linked to impaired reproductive health. For instance, women with Type 1 diabetes (typically treated with exogenous insulin) are more likely to exhibit ovarian dysfunction, and those taking a higher daily dose of insulin have an increased chance of earlier menopause [261,262]. An early diagnosis of Type 2 diabetes, which is closely tied to obesity and high endogenous insulin, is also predictive of earlier menopause [261,263–265]. Elevated insulin is a cardinal feature of PCOS that aggravates its reproductive pathophysiology, by augmenting testosterone production and bioavailability as well as inhibiting follicular growth and maturation [88,89]. Therefore, while insulin signaling is essential for metabolic and reproductive functions, preventing insulin excess could have promising therapeutic potential [207].

## Glucose-dependent insulinotropic polypeptide (GIP) and glucagon-like peptide 1 (GLP-1)

Incretins are metabolically active gut hormones released promptly after food consumption. Seminal work in the 1960's pointed to the existence of factors that heighten insulin levels in response to ingestion [266–268]. Glucose-dependent insulinotropic polypeptide (GIP) was isolated in 1971 [269] and shown to potentiate insulin levels in response to intestinal absorption of nutrients such as glucose [270]. Glucagon-like peptide 1 (GLP-1) was later identified as another potent insulinotropic hormone [271]. GLP-1 also promotes the proliferation of β-cells [272,273] and prevents their apoptosis [274]. In addition, GLP-1 contributes towards maintaining glucose homeostasis by lowering food intake, glucagon secretion, and endogenous glucose production [275–277].

GIP and GLP-1 are produced by intestinal enteroendocrine cells. GIP is secreted by the K-cells of the small intestine in response to the ingestion and absorption of glucose, lipids, and high levels of amino acids [270,278–281]. GLP-1 is secreted by the large intestine and the L-cells of the small intestine [282,283]. GLP-1 is the post-translational cleavage product of the *proglucagon* gene, and is stimulated by monosaccharides such as glucose, fructose, and galactose [284,285], as well as dietary lipids [286,287] and amino acids [288–291]. Upon their release into circulation, the incretins bind to their respective G-coupled receptors (GIPR and GLP-1R), which in pancreatic β cells stimulate insulin exocytosis by inducing a rise in intracellular cAMP and calcium levels [292,293]. The insulinotropic effects of incretins are largely mediated by β cells, but incretin receptors have a broad distribution across many tissues, including in the hypothalamus [292–294]. GLP-1 produced within the brain also appears to contribute to its central effects [292,295].

Despite higher circulating levels, the insulinotropic effects of GIP dwarf in comparison to GLP-1, which is widely popularized as a therapeutic target. GLP-1 can normalize blood glucose levels in Type 2 diabetes patients [296] and promote weight loss [297]. However, GIP and GLP-1 are enzymatically inactivated after secretion, and the rapid degradation of GLP-1 limits its therapeutic potential [298]. Efforts have now shifted to using GLP-1 receptor agonists [299–301] or dual GIP- and GlP-1- receptor agonists [300,302,303]. Incretin-based therapy has expanded to target fatty liver disease [304,305], kidney disease [306], neurodegenerative diseases [307,308] and reproductive disorders such as PCOS [309,310].

### Incretin hormones and the female reproductive system

The incretin hormones might elicit indirect, insulin-mediated impacts on female reproductive function due to their insulinotropic nature, but they also directly affect reproduction. Mice deficient in either GLP-1R or GIPR exhibit disrupted estrous cycling, reduced fertility, and smaller litter sizes [311], and GLP-1R knockout additionally leads to delayed puberty in female mice [312]. GLP-1 may exert many of these reproductive effects through central actions. Both hypothalamic *GLP-1r* expression and plasma GLP-1 concentrations vary across estrous phases in rats, and either central or peripheral administration of GLP-1 increases the preovulatory LH surge [313,314]. Intracerebral GLP-1 also synchronizes the onset of puberty, and improves implantation rates, birthing rates, and mature follicle numbers [314]. Changes in gonadotropin secretion appear to be due in part to GLP-1 positively regulating GnRH release. Early evidence in a rat hypothalamic cell-line pointed to GLP-1 promoting GnRH release via intracellular cAMP signaling [315], and subsequent work implicated the involvement of Kiss1 neurons [314,316,317] and GABAergic signals [318] in bolstering the direct effects of GLP-1 on GnRH neurons. While there is less known about the role of GIP, GIPR is expressed in the murine hypothalamus [319] and pituitary [311], and intracerebroventricular GIP administration decreases plasma FSH levels in rats [320]. Receptors for both GLP-1 and GIP are also expressed in the rodent ovary [311,321], and both of these incretins suppress progesterone synthesis in the presence of FSH [321]. Incretins have also been detected in human follicular fluid, and tend to be higher in the follicular fluid of obese women (particularly GLP-1) [322].

There has been some investigation into the therapeutic effects of incretin receptor agonists for female reproductive disorders. For instance, GLP-1 receptor analogues can mitigate the ovarian inflammation, fibrosis, oxidative stress, and AMH reduction that is induced in a rat model of diabetes [323]. In animal models of PCOS, incretin receptor agonists improve ovarian morphology and gonadotropin levels [324,325]. Incretin analogues are also being applied in clinical practice with PCOS patients. They can effectively alter steroid hormone levels [326–329], decrease body weight and enhance metabolic health [330–333], regularize menstrual cycling [329,331,334], and improve pregnancy rates and outcomes [331,333]. However, in general there is a paucity of information on the details and mechanisms by which anti-obesity pharmaceuticals such as incretin receptor agonists affect the female reproductive system [309,335].

## Growth hormone (GH)

First isolated in the 1940s, growth hormone (GH) was defined by impacts on longitudinal growth largely driven by a promotion of bone growth in children and adolescents [336,337]. However, it is now known that GH also has a broader reach in regulating energy balance, including effects on puberty timing, reproductive function, insulin resistance, metabolic fuel selection, lipolysis, hepatic glucose production, protein synthesis, muscle building, and immune function [337–339]. In general, GH is anabolic in nature. It stimulates an increase in lean body mass under energy-replete conditions, and preserves lean body mass and carbohydrate stores during fasting by promoting lipid usage [337]. GH elicits its physiological outcomes through a combination of indirect, IGF-1-mediated effects and direct intracellular signaling via the widely expressed GH receptor, which activates Janus kinase 2 (JAK2)-signal transducers and activators of transcription (STAT) as well as other signaling cascades [339]. Since GH promotes a rise in circulating and locally produced levels of the insulin-like growth factor IGF-1, it can be difficult to mechanistically distinguish between direct GH effects and ancillary effects carried out through IGF-1 signaling [337].

GH is secreted in a pulsatile manner by somatotroph cells in the anterior pituitary gland, with levels and patterns that depend on age, sex, and energy balance [340–342]. Hypothalamic-produced GH-releasing hormone stimulates its secretion while somatostatin inhibits it [340,343]. Additionally, ghrelin potently stimulates GH secretion, and both estrogens and androgens promote GH release [340,343–348]. Outside of the brain, *GH* mRNA is also expressed in peripheral tissues, including in the uterus, mammary glands, and ovaries; locally produced GH likely has local autocrine and/or paracrine effects, rather than traveling through circulation [349]. In general, circulating GH is higher in females than males, and levels rise in response to puberty, sleep, exercise, and fasting, whereas GH is decreased in response to elevated blood glucose, glucocorticoids, and aging [340,348]. Circulating GH levels peak at puberty and decline steadily afterwards, with only residual levels detectable at age 50 [350–354].

### Growth hormone and the female reproductive system

Having sufficient GH is important for multiple facets of female reproductive competency. Women with GH deficiency have delayed menarche, fewer children, reduced uterine volume, low prolactin levels, and higher FSH [355,356]. Similarly, GH-deficient rodents have a later onset of puberty, smaller litter sizes, delayed parturition, irregular estrous cycles, and fewer corpora lutea and follicles [357–360]. GH replacement therapy has therapeutic potential in GH-deficient infertile women [361], and also improves ovulation rates and embryo implantation rates in women undergoing IVF when combined with gonadotropin treatment [362,363]. Genetically engineered GH-overexpressing animals have increased ovarian weights as well as higher ovulation rates and implantation sites, but also exhibit lower mating rates and reduced offspring survival [364,365].

In addition to regulating pubertal growth, GH is implicated in controlling the timing of puberty. Puberty is delayed in mammals with GH deficiency, although the fact that they can reach sexual maturation indicates that GH is not a requirement [366,367]. GH transgene expression expedites puberty in mice [365], and GH treatment in GH-deficient children stimulates an earlier age of puberty [368]. GH affects GnRH release [369,370], and GH and/or IGF-1 signaling in GnRH neurons or Kiss1 neurons could play a role in the activation of pulsatile hypothalamic GnRH secretion linked to the onset of puberty (reviewed in [366]). However, the start of ovarian steroidogenesis and consequential rise in steroid hormones is instrumental in promoting the steep elevation in GH levels during puberty, making it difficult to tease apart these causal relationships [366,367]. Ultimately, it is most likely that interactions between GH and the HPG axis are bidirectional during the complex endocrine shifts of puberty, and GH may be one of the integrated endocrine signals that conveys whether nutrient levels are sufficient for puberty to proceed [366,367].

At the level of the anterior pituitary, it is noteworthy that there are interactions between GH-producing somatotroph cells and gonadotropin-producing gonadotroph cells (reviewed in [367]). Therefore, GH likely exerts some of its reproductive effects within the pituitary itself, through such means as influencing the secretion of LH and FSH [367]. However, GH plays a more apparent role in regulating reproductive function through its ovarian actions.

In the ovary, GH is involved in governing gametogenesis, gonadotropin sensitivity, follicle survival, and the preservation of tissue health [371]. GH receptors are present in the oocytes and granulosa cells of antral follicles [372], and levels are significantly decreased in lower-quality oocytes of aging women [373]. *In vitro* studies of goat oocytes reveal that GH treatment stimulates early antral follicle development, promotes fertilization, development of healthy oocyte-cumulus complexes and growth of a healthy embryo [374]. Similarly, in canine oocytes GH acts alongside FSH to promote antrum formation, resulting in improved follicular viability [375]. Other studies have suggested that GH prevents follicular apoptosis via IGF-1 and the PI3K/AKT signaling pathway [376,377]. *In vivo* work has shown that GH may improve ovulation rates by increasing the number of superovulated oocytes reaching meiosis II [378]. *In vitro* studies point to a similar trend of GH supplementation leading to increased meiotic progression rates [374], and improved nuclear maturation in rodent [379], dog [380], sheep [381], bovine [382], equine [383], and human [384] oocytes. These effects may be partially mediated by cumulus cells. GH stimulates proliferation and inhibits apoptosis of cumulus cells [385,386], and regulates the expression of the gap junction proteins that allow oocytes to exchange nutrients with surrounding cumulus cells [385,387]. Murine oocytes cultured with GH form thecal layers that are rich in mitochondria and rough ER, implicating an additional role in theca cell proliferation [388].

The clinical potential for GH also stems from its role in improving uterine receptivity to incoming embryos. GH-stimulated upregulation of IGF-1 mediates estrogen-related improvements in endometrial receptivity and increase uterine thickness across several species [389–393]. Although more contentious, human studies also suggest benefits of GH therapy among female IVF and embryo transfer patients, especially if they have endocrine disorders or are overweight/obese [394,395]. GH concentrations are higher in the follicular fluid of oocytes that result in successful pregnancy [396,397], and GH supplementation increases oocyte yield as well as rates of pregnancies and live births with IVF [398,399]. Thus, GH is becoming an increasingly important compound-of-interest in assisted reproductive technologies [363,400–405].

## Ghrelin

Identified in 1999 as the endogenous ligand of the growth hormone secretagogue receptor (GHSR) [406], acetylated ghrelin is a gastric hormone involved in sensing nutrient availability and coordinating meal anticipation, which complements its stimulation of growth hormone secretion and other metabolic effects [407]. Dubbed the ‘hunger hormone’ for its appetite-boosting effects [408], acetylated ghrelin is in fact a multi-faceted hormone that also stimulates gastric acid secretion and gut motility, promotes adiposity, decreases insulin sensitivity, and modulates glucose and lipid metabolism [407,409]. Ghrelin and its receptor are both widely expressed in human tissues, including in reproductive and endocrine organs [410], but most circulating ghrelin originates from enteroendocrine cells of the stomach [411]. Levels rise before meals and during fasting, largely due to neural regulation of gastric ghrelin secretion; conversely, there is a postprandial drop in ghrelin in response to nutrients and bitter compounds in the gastrointestinal system as well as input by hormones such as insulin and leptin [408,409,411,412]. Circulating ghrelin exists in two distinct forms due to its enzyme-catalyzed acetylation. Less than 10% is acetylated and capable of binding to the GHSR, while des-acylated ghrelin functionally antagonizes acetylated ghrelin and may also have independent effects [407,409].

### Ghrelin and the female reproductive system

As an orexigenic hormone that signals nutrient insufficiency, ghrelin is generally a negative modifier of female reproduction. Women with amenorrhea associated with intense exercise or anorexia have higher levels of ghrelin [413–415]. Ghrelin levels decline during childhood and into puberty [416], and pubertal onset in female rats is delayed by high doses of ghrelin [417–419]. High ghrelin or ghrelin analog treatment reduces rates of ovulation, pregnancy, fertilization, and embryo implantation in mice, and suppresses ovine embryo development [420–423]. Interestingly, ghrelin levels increase with age and over the menopausal transition [412,424,425], which may contribute to postmenopausal shift in metabolic health [426].

Central *in vivo* effects of ghrelin include decreasing GnRH secretion and pulsatility, as well as lowering LH and/or FSH [415,418,427–431]. The GHSR is expressed in regions of the hypothalamus [432,433], Kiss1 neurons [434], and pituitary gonadotrophs [435]. Although GnRH neurons themselves do not express this receptor, ghrelin might act via upstream neuronal regulators to suppress GnRH release [221]. In contrast, ghrelin can stimulate LH secretion by pituitary tissue *in vitro*, pointing to an opposing, tissue-specific mode of action that might depend on such factors as age, sex, and interacting gonadal inputs [417,418,436].

Ghrelin also exerts direct ovarian effects. Ghrelin expression has been documented in the ovaries of species ranging from chicken to human [437–443], including in oocytes, corpus lutea, and stromal cells [437,439,440]. Ghrelin injections lower estrogen and progesterone levels in female rats [444], and it acts directly through GHSR of corpus luteal cells to reduce progesterone secretion [445,446], pointing to a role in regulating steroidogenesis. Ghrelin may also contribute toward repressing follicle maturation: ghrelin administration leads to greater numbers of small follicles coupled with fewer corpus lutea in rat ovaries [447], whereas mice lacking endogenous acetylated ghrelin have a decrease in small follicles [448]. Generally, elevated ghrelin suppresses female reproductive functions both centrally and peripherally, which is consistent with a message of energy depletion.

## Leptin

Leptin, an indicator of stored fuel levels in adipose tissue, is involved in regulating long-term energy balance by influencing parameters such as energy expenditure, appetite, and reproductive function. Leptin is a peptide hormone principally secreted by adipocytes in white adipose tissue, though it can also be produced by other tissues such as the placenta, stomach, and skeletal muscle [449–453]. Serum leptin and adipocyte expression of the leptin gene (*LEP* or *OB*) are proportional to adipose tissue mass, with levels that generally rise with obesity and fall with weight loss [454]. *LEP* expression and circulating leptin are also affected by short-term energy imbalances, cytokines, the sympathetic nervous system, and other hormones such as insulin, glucocorticoids, and gonadal steroids [455]. In both humans and rodents, leptin levels are higher in females than in adiposity-matched males (particularly for premenopausal women); this is likely due to the regulation of leptin production by estrogens and testosterone, as well as sexual dimorphism in adipose tissue distribution [456–460]. Although leptin is not alone in controlling energy balance, its crucial role is evidenced by the excessive obesity, hyperphagia, and infertility of the leptin-deficient *ob/ob* mouse [461–463]. Similarly, rare mutations causing congenital leptin deficiency or leptin resistance in humans are associated with rapid weight gain, severe obesity, low gonadotropin levels, and delayed or absent puberty [464–467].

In response to elevated leptin—which indicates that energy balance has tipped towards abundant metabolic fuel stores—the hypothalamus induces a series of physiological processes that boost satiety and energy expenditure [467,468]. However, obesity is often coupled with both increased leptin levels and leptin resistance, which diminishes its effectiveness in promoting weight loss [467]. Low leptin levels correlate with reduced adiposity, which is interpreted by the hypothalamus as an energy deficit that requires neurological and physiological changes to promote food intake while reducing energy expenditure to restore energy balance [467].

The canonical leptin signaling pathway involves activation of JAK2 and phosphorylation of the transcription factor STAT3. However, due to pathway cross-talk leptin also activates other signal transduction cascades, such as the PI3K and MAPK pathways [468]. Only the full-length, long-form isoform of the leptin receptor has the intracellular domains required for signal transduction [469,470]. Although particularly abundant in the hypothalamus, there is nearly universal tissue distribution of leptin receptors, including expression of the long-form receptor in many brain regions and in the uterus and ovaries [469–472]. This highlights the fact that leptin signaling has a wide breadth of effects. In addition to its trademark impacts on appetite and energy expenditure, leptin is also involved in controlling lipolysis, immune function, angiogenesis, bone formation, and reproduction [468].

### Leptin and the female reproductive system

Leptin is a fundamental regulator of female reproductive function that affects processes ranging from steroidogenesis and ovulation to puberty and pregnancy. Negative energy balance leads to decreased leptin, amenorrhea, and subfertility [473,474], and leptin administration in women with hypothalamic amenorrhea is sufficient to restore their menses and fertility, raise serum estradiol, and increase the number of dominant follicles [475–477]. Exogenous leptin also restores the fertility of *ob*/*ob* mice independent of body weight effects, by restoring HPG axis functioning [478,479]. Similarly, daily leptin injections in leptin-deficient children correct pubertal timing and gonadotropin pulsatility [480]. In rodents, leptin aids in pubertal activation of the HPG axis [481,482], although it alone cannot trigger puberty alone [478,483–486]. Low doses of leptin increase LH and FSH levels in mice [487], induce ovulation in an LH-dependent manner [488], and stimulate meiotic progression of bovine oocytes [489].

Leptin exerts some of these effects through indirect modulation of GnRH neurons and the pituitary. Leptin receptors are undetectable in murine GnRH neurons, and GnRH neuron-specific leptin receptor deletion does not affect fertility or puberty onset. However, mice lacking leptin receptors in all forebrain neurons have delayed puberty, severe infertility, and a suppressed estradiol-stimulated LH surge [490]. Leptin likely impacts GnRH release via upstream neuronal inputs [221], such as Kiss1 neurons [490–493]. It may also elicit direct effects on the pituitary [494], since cultured pituitary tissue dose-dependently releases LH, FSH and prolactin in response to leptin exposure [495].

There is also a relationship between leptin and the ovarian steroid hormones. *LEP* is expressed by granulosa, cumulus, and oocyte cells, with leptin protein detectable in mature follicles and follicular fluid [496,497]. Human and rat ovaries express leptin receptors on their theca, granulosa and interstitial cells [471,496–499], thus acting as target sites for leptin to regulate steroidogenesis. *In vitro* studies of human [499,500], bovine [501], and rat [502–504] cells or tissues have demonstrated that leptin attenuates steroid hormone production. Interestingly, there are reports of circulating leptin changing across the menstrual cycle, with a rise from menses into the luteal phase and a mid-cycle peak corresponding with the LH surge; leptin therefore shows some synchronicity with estradiol, progesterone, testosterone, and LH [475,505–507]. Some of these rhythmic changes may be due to leptin production by ovarian structures such as the corpus luteum [508,509]. However, leptin cyclicity during the menstrual cycle is controversial, with other studies reporting no differences [510–512].

During pregnancy, there is a two-fold increase in circulating leptin levels [513,514], due to both increased maternal adiposity and leptin secretion by the placenta [452]. As pregnancy progresses, the rise in leptin induces central resistance to its appetite-suppressing effects, and leptin takes on modified roles that include support of blastocyst formation, implantation, placentation, and human chorionic gonadotropin production [515–518]. Supraphysiological levels of leptin are associated with pregnancy disorders such as preeclampsia [519–524] and gestational diabetes [525]. Thus, leptin is involved in optimising and maintaining many aspects of female reproductive health and function.

## Adiponectin

In addition to leptin, adipocytes also secrete another signaling molecule in large quantities: adiponectin. First discovered and characterized in 1995–1996, adiponectin is a 244-amino acid protein [526–529] with insulin-sensitizing [530,531], anti-inflammatory [532], anti-atherogenic [532–535], and cardioprotective [536,537] properties. Adiponectin is primarily secreted by adipocytes, but has been detected in other tissues, including the brain [538,539], gonads [540,541], and placenta [542]. In contrast with leptin, adiponectin levels are inverse to adiposity; adiponectin is lower among obese individuals [543–545], and restricting caloric intake can increase circulating adiponectin [546]. Testosterone also reduces adiponectin secretion [547], which may contribute toward the lower levels in men compared with women [543,544]. There are no apparent repercussions of menopause, estrogen therapy or ovary removal for adiponectin levels [548–550].

Adiponectin signals through binding to adiponectin receptors 1 and 2 (AdipoR1 and AdipoR2), which are found abundantly among several tissues, but especially in the skeletal muscle and liver [551,552]. Ligand binding leads to a number of downstream signaling responses, including AMPK activation, mTOR inhibition, stimulation and cross-talk with the insulin/IGF-1 signaling pathways, and interactions with other signal transduction adaptor proteins [196]. These adiponectin-induced signaling cascades in central and peripheral tissues induce metabolically important responses. Exogenous adiponectin administration increases blood insulin levels *in vivo* [553], and promotes insulin gene expression and secretion *in vitro* [554]. Adiponectin-deficient mice develop hepatic insulin resistance and hyperglycemia, and are more sensitive to diet-induced metabolic dysfunction [530]. In addition, adiponectin-deficient female mice are subfertile, with altered menstrual cycles, altered gonadotropin profiles, reduced ovulation and a greater number of atretic follicles [555]. This points to a role in reproductive regulation.

### Adiponectin and the female reproductive system

Adiponectin elicits both central and peripheral reproductive effects. Adiponectin receptors are found throughout the hypothalamus in a variety of species [556–558], and adiponectin is present in cerebrospinal fluid [557,559–562], which suggests a potential route of entry into the brain. Consistent with a function in promoting energy preservation, adiponectin suppresses GnRH secretion and inhibits *Kiss1* gene expression by activating AMPK in a hypothalamic cell line [563,564], and it attenuates activity of a subpopulation of mouse GnRH neurons via AMPK activation [565]. Humans also express adiponectin and its receptors on pituitary cells, including gonadotrophs [566]. Adiponectin can reduce basal and GnRH-stimulated LH levels and GnRH receptor expression in cultured rodent pituitary cells [538,567], but reported effects are not consistent for pigs [568] or non-human primates [569].

In the ovary, adiponectin signaling influences oocyte maturation as well as the production and release of steroid hormones. Adiponectin and adiponectin receptors are expressed in ovarian theca cells, granulosa cells, oocytes, and corpus lutea [541,570–572], and levels appear somewhat responsive to gonadotropins [573–575]. Adiponectin regulates the expression of genes encoding steroidogenic enzymes and gonadotropin receptors, augments the IGF-1-stimulated release of progesterone and estradiol, and decreases androgen levels [541,570,576–579]. Adiponectin is also implicated in promoting oocyte meiotic maturation and early embryo development [572,580–583]. The mechanisms by which adiponectin exerts these ovarian effects have not been fully defined, but may involve interactions with the insulin/IGF-1 MAPK/ERK signaling pathway [541,570,576,579,581,582].

Low adiponectin (which can signify overabundant energy stores) is linked to reduced female reproductive health. Adiponectin levels are positively correlated with levels of the ovarian reserve biomarker AMH [215,584], and obesity corresponds with both low adiponectin and low AMH, among other endocrine changes [585]. Women with PCOS also have significantly lower adiponectin levels that correlate with lower metabolic health, compared with individuals matched for body mass index [586,587]. In addition, a decreased proportion of theca cells express adiponectin receptors in polycystic ovaries [588]. Low serum adiponectin or a low ratio of follicular fluid:serum adiponectin has been associated with unsuccessful IVF outcomes [589], higher rates of implantation failures [590], and low oocyte retrieval [591]. Therefore, adiponectin is yet another metabolic hormone at the nexus of metabolic health and reproductive function.

## Conclusions and future perspectives

Research related to female reproductive health is disproportionately underfunded [592–595], with consequential impacts on the well-being of half the global population. Moreover, despite the existence of sex differences in the prevalence, pathophysiology, and responses to treatment of metabolic disorders such as Type 2 diabetes [9,10,596–598], a significant underrepresentation of female participants and female animals in metabolic health studies has persisted in recent decades [599–604]. As a result, each of these fields alone holds substantial knowledge gaps—to say nothing of the gaps in knowledge that exist at the interface of metabolic health and female reproductive health.

We believe that there are many critical questions at the junction of metabolism and reproduction. For instance:

- While this review highlights effects of metabolic hormones on reproductive function, lines of communication between metabolic tissues and the reproductive system are bidirectional, and merit further study. For example, FSH was recently shown to regulate insulin secretion via FSH receptors expressed in pancreatic islets [605], and gonadal steroid hormones contribute towards sexual dimorphism in energy partitioning and metabolic homeostasis [8–10,606].
- Exogenous hormonal contraceptives cause metabolic changes [607–609], and conversely, the presence of diabetes, obesity, and/or other metabolic disorders has implications for the systemic impacts of hormonal contraceptives [610,611]. Research into these intertwining effects is complicated by the heterogeneous nature of disorders such as Type 2 diabetes or polycystic ovary syndrome, in addition to wide variety in hormonal contraceptive formulations, and interplay of factors such as age, ethnicity, genetics, environment, and duration of contraceptive use [608,612].
- Similar complications affect investigations of interactions between metabolic health and hormone replacement therapy, or between metabolic health and menstrual cycle characteristics, but these challenges should not preclude exploring such fundamental biomedical and biological topics.
- Puberty, pregnancy, and perimenopause are defined by changes to the female reproductive system, and all three life stages also feature marked metabolic changes. For instance, insulin resistance and β-cell mass are transiently elevated during puberty [613–616] and during pregnancy [617–620], and the incidence or severity of metabolic syndrome increases significantly during perimenopause [621,622]. Therefore, it seems especially pertinent to understand the relationships between nutritional status, metabolic health, and female reproductive function during these transitional periods.

The high energetic requirements of supporting reproduction mean that the signaling systems communicating food intake, metabolic fuel stores, and energy levels play an integral role in regulating reproductive function. Consequently, impaired metabolic health has repercussions for female reproductive health that extend beyond fertility or fetal effects. Changes to nutritional status induce a suite of responses, and it is necessary to consider the context of a broad landscape of nutrient- and energy-responsive signaling systems instead of focusing on isolated hormones under specific conditions. For instance, food ingestion or a surplus of energy stores generally leads to increased levels of insulin, GIP, GLP-1, and leptin, together with suppression of growth hormone, ghrelin, and adiponectin; energy deficits tend to cause the opposite endocrine shifts (Figure 1). Each of these hormones can cause their own effects within the reproductive axis, in addition to generating signaling pathway cross-talk and interplay with other hormones. Moreover, the precise effects of each hormone might vary depending on nutritional conditions and interacting signaling factors. Delineating the complexities of these mechanistic relationships is essential for understanding how metabolic disorders or energy imbalance deregulates female reproductive health.

## Abbreviations

AdipoR: adiponectin receptor
AdipoR1 and AdipoR2: adiponectin receptors 1 and 2
AMH: anti-müllerian hormone
AMPK: AMP- activated protein kinase
FSH: follicle-stimulating hormone
GH: growth hormone
GHSR: growth hormone secretagogue receptor
GIP: glucose-dependent insulinotropic polypeptide
GLP-1: glucagon-like peptide 1
GnRH: Gonadotropin-releasing hormone
HPG: hypothalamic-pituitary-gonadal
IGF-1: insulin-like growth factor 1
InsR: insulin receptor
JAK2: janus kinase 2
Kiss1: kisspeptin
LH: luteinizing hormone
MAPK: mitogen-activated protein kinase
mTOR: mechanistic target of rapamycin
PCOS: polycystic ovary syndrome
PI3K: phosphatidylinositol 3-kinase
STAT: signal transducers and activators of transcription
